# Antimicrobial and Antibiofilm Activity of Chitosan Nanoparticles Against *Staphylococcus aureus* Strains Isolated from Bovine Mastitis Milk

**DOI:** 10.3390/pharmaceutics17020186

**Published:** 2025-02-02

**Authors:** Carlos Alarcón Godoy, Iván Balic, Adrián A. Moreno, Oscar Diaz, Carla Arenas Colarte, Tamara Bruna Larenas, Alexander Gamboa, Nelson Caro Fuentes

**Affiliations:** 1 Centro de Investigación Austral Biotech, Facultad de Ciencias, Universidad Santo Tomás, Avenida Ejército 146, Santiago 8370003, Chile; calarcon8@santotomas.cl (C.A.G.); carenas8@santotomas.cl (C.A.C.); tbruna@santotomas.cl (T.B.L.); 2 Área Prioritaria de Investigación (API3), Programa Fitogen, Departamento de Acuicultura y Recursos Agroalimentarios, Universidad de Los Lagos, Osorno 5311157, Chile; ivan.balic@ulagos.cl (I.B.); oscar.diaz@ulagos.cl (O.D.); 3 Centro de Biotecnología Vegetal, Facultad de Ciencias de la Vida, Universidad Andres Bello, Santiago 8370146, Chile; adrian.moreno@unab.cl; 4 Facultad de Química y Biología, Universidad de Santiago de Chile, Av. Libertador Bernardo O’Higgins 3363, Estación Central, Santiago 9170022, Chile

**Keywords:** bovine mastitis, antibiofilm, chitosan nanoparticles, antimicrobial, *Staphylococcus aureus*

## Abstract

Background/Objectives: Bovine mastitis (BM), a prevalent and economically burdensome bacterial infection affecting dairy cattle, poses a significant challenge to the dairy industry. The traditional approach to combating BM, relying heavily on antibiotics, faces growing concerns due to the increasing antibiotic resistance exhibited by pathogens. The objective of this study was to evaluate and determine the antimicrobial and anti-biofilm potential of chitosan nanoparticles (NQo) on *S. aureus* strains isolated from milk samples obtained from dairy areas in southern Chile from cows diagnosed with BM. Methods: NQo were synthesized using the ionotropic gelation method and thoroughly characterized by transmission electron microscopy (TEM) and dynamic light scattering (DLS). Results: The NQo exhibit a robust positive charge (Z-potential of +55.4 ± 2.5 mV) and an exceptionally small size (20.3 ± 3.2 nm). This unique combination of properties makes NQo particularly well-suited for targeting and interacting with bacterial pathogens. To assess the effectiveness of NQo against BM, we conducted a series of experiments using a *Staphylococcus aureus* strain isolated from milk samples of cows diagnosed with BM in southern Chile. NQo demonstrated a remarkable ability to inhibit bacterial proliferation and effectively modulate biofilm formation in the *S. aureus* strains. Furthermore, the performance of NQo in comparison to established antibiotics like ampicillin and gentamicin strongly suggests that these nanoparticles hold immense potential as an attractive alternative for the control, prevention, and/or treatment of BM. Conclusions: NQo exhibit both antimicrobial and antibiofilm activity against a clinically relevant BM pathogen. Further investigations are necessary to develop a hydrogel formulation optimized for effective delivery to the target diseased tissue.

## 1. Introduction

Bovine mastitis (BM) is a severe epidemic and one of the most significant challenges confronting the dairy industry in Chile and worldwide [1]. Globally, this disease imposes economic losses estimated between USD 19.7 and 32 billion annually [2]. BM is primarily caused by bacterial infections from various strains [3,4], including *Staphylococcus aureus*, and poses a significant threat to cattle health and dairy production [5]. This disease spreads rapidly within herds, facilitated by close contact and shared milking equipment, making containment challenging [6]. It leads to unnecessary animal suffering, substantial treatment expenses, and, in severe cases, mass culling of livestock. These consequences drastically reduce production and profitability in the dairy sector [7]. Currently, this infection is treated with antibiotics, which are often overused in the dairy industry. This excessive use drives a natural process in bacteria known as antibiotic resistance, diminishing the effectiveness of conventional treatments [5]. As a result, the disease spreads more rapidly, exacerbating the problem and creating significant long-term challenges [8,9]. Studies and in vitro tests on bovine samples have demonstrated that the active ingredient formulated with chitosan nanoparticles (NQo) possesses antibacterial properties that are equally as effective as conventional antibiotics [10]. Chitosan is a heteropolysaccharide obtained from chitin [11]. It consists of glucosamine and *N*-acetylglucosamine units linked by linear β-1,4-linkages [12]. Ionotropic gelation, as described by Calvo et al. [13], is a method widely recognized for its efficiency in producing NQo with high stability and biocompatibility, making it ideal for biomedical applications, such as drug delivery. Ionotropic gelation involves the cross-linking of chitosan with anionic agents (example, tripolyphosphate) under mild conditions, which preserves the active properties of the material while allowing for precise control over particle size and morphology [14,15]. This synthesis approach ensures the consistent quality of NQo, enhancing their antibacterial properties [10]. Similarly, chitosan nanoparticles have demonstrated antibiofilm properties, effectively targeting a bacterial defense mechanism that conventional antibiotics cannot address [10]. The use of chitosan nanoparticles would offer significant advantages, such as reduced adverse effects on cattle and faster recovery of milk production. This would not only improve overall animal health and welfare but also minimize the economic impact on the dairy industry [16]. Large-scale farmers benefit from improved productivity and cost efficiency, while small-scale farmers gain access to an affordable and effective treatment option that helps maintain the sustainability of their operations [16]. By addressing the challenges of bacterial resistance and ensuring healthier livestock, this approach holds promise for improving dairy farming practices across diverse scales. Milk produced by cows undergoing antibiotic treatment, as well as milk produced for 3 to 4 days following the last treatment, must be discarded to prevent antibiotic residues from entering the food supply [17]. This discarded milk represents a significant financial burden, accounting for 53% to 80% of the direct costs associated with each day of treatment [8]. These losses not only strain the profitability of dairy operations but also contribute to the overall economic challenges of managing bovine mastitis [18]. To address this problem, we propose that the synthesis of very small chitosan nanoparticles will be more effective than conventional antibiotics in eradicating biofilms. The objective of this study was to evaluate and determine the antimicrobial and antibiofilm potential of NQo on *S. aureus* strains isolated from milk samples obtained from dairy areas in southern Chile from cows diagnosed with BM. The novelty of our findings resides in the successful synthesis of remarkably small chitosan nanoparticles, significantly smaller than those reported in previous studies [19]. Furthermore, these nanoparticles exhibit a high Z-potential, which enhances their capacity to disrupt bacterial cell walls [20].

## 2. Materials and Methods

### 2.1. Materials

Low-molecular-weight chitosan with a degree of deacetylation (DD) ≥ 75 and Sodium tripolyphosphate were acquired from Sigma–Aldrich Inc. (St. Louis, MO, USA). Acetic acid (Merck, Darmstadt, Germany) *S. epidermidis* ATCC 12228 (MedicaTec, S.A., Santiago, Chile). Baird Parker agar, Trypto-Casein Soy Broth, and Congo red agar (Merck, Darmstadt, Germany) were used. All other chemicals used were analytical grade.

### 2.2. Synthesis of Chitosan Nanoparticles (NQo)

Chitosan nanoparticles were synthesized using an ionotropic gelation method, as described by Calvo et al. [13], with certain modifications [21]. Briefly, 0.3% (*w*/*v*) low-molecular-weight chitosan is prepared in 0.1 M acetic acid and stirred for 24 h. The Qo solution is mixed in a 2:1 (*v*/*v*) ratio with 0.1% (*w*/*v*) tripolyphosphate (TPP) solution and added dropwise using an infusion pump (KDS200, KD Scientific©, Holliston, MA, USA) with a drip rate of 1.8 mL/min, with constant magnetic stirring at room temperature. The suspension obtained is centrifuged at 21,000× *g* for 30 min at 14 °C (HermLe model Z32K, Wehingen, Germany). The supernatant is stored for experiments.

### 2.3. Characterization of NQo

#### 2.3.1. Determination of Size, Zeta Potential, and Polydispersity Index

The surface charge and size distribution of the nanoparticles were determined using a Zetasizer Nano ZS-90 (Malvern Panalytical Ltd., Malvern, UK). For the analysis, 1.0 mL of the nanoparticle suspension was diluted tenfold with water and subjected to sonication for 1 h [14]. Subsequently, the sample was meticulously transferred into a folded polystyrene capillary cell (model ZS90 Malvern Panalytical Ltd., Malvern, UK). Measurements were conducted under standard conditions (temperature of 25 °C, and a laser wavelength of 633 nm) [22].

#### 2.3.2. Transmission Electron Microscopy (TEM)

Particle images were captured using transmission electron microscopy (TEM) with a Philips Tecnai 12 Bio Twin microscope (Eindhoven, The Netherlands), providing detailed visualization of the nanoparticles’ morphology and structure. The process involved depositing a droplet of the nanoparticle suspension onto a coated copper grid (SPI Supplies, Inc., West Chester, PA, USA). The grid was then allowed to dry thoroughly before proceeding with transmission electron microscopy (TEM) analysis. The particle size from the images was analyzed using ImageJ software (version 1.52). Histogram generation and cumulative distribution analysis were conducted using Origin 8 software (version 8.0773).

### 2.4. S. aureus Strains from Mastitis Bovine Milk Samples

The *S.aureus* strains were isolated from milk samples collected from symptomatic Holstein Friesian cows in Los Muermos, located in the X Región de Los Lagos, Chile. These cows were diagnosed with clinical mastitis by veterinary professionals. To determine the presence of bovine mastitis in cows, the specimens showed the characteristics described by Rivera et al., (2020) [10]. The following flowchart outlines the procedural framework for the isolation process.


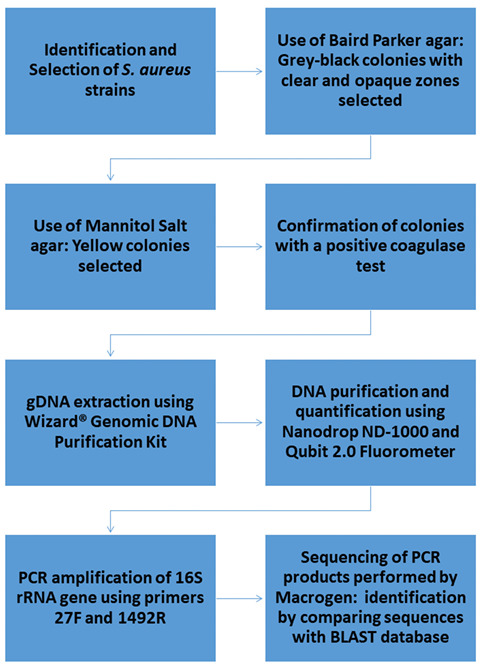


### 2.5. Quantification of Biofilm Production Capacity Using Crystal Violet

Overnight cultures of *Staphylococcus aureus* strains were diluted 1:100 in fresh Trypto-Casein Soy Broth (TSB) to achieve a concentration of 1 × 10^7^ CFU/mL. Aliquots of 200 µL were dispensed into individual wells of a 96-well polystyrene microtiter plate and incubated at 37 °C for 24 h. Biofilm formation was then assessed using a crystal violet staining method [23]. The wells were stained with a 1% (*w*/*v*) crystal violet solution for 5 min. After three thorough washes with distilled water, the stained biofilm was re-suspended in 33% (*v*/*v*) glacial acetic acid. The optical density (OD) was then measured at 570 nm using an Infinite m200 Pro TECAN Plate Reader (Tecan AG Männedorf, Switzerland). To evaluate the impact of NQo on biofilm production, the initial bacterial dilutions were co-incubated with varying concentrations of NQo.

Categorization of isolates based on biofilm-forming capacity.

The following criteria were used for biofilm gradation in clinical isolates:

OD_cut_ = OD_avg_ of negative control + 3 × standard deviation (SD) of ODs of negative control.

1. OD ≤ OD_cut_ = Non-biofilm-former (NBF).
2. OD_cut_ < OD ≤ 2 × OD_cut_ = Weak biofilm-former (WBF).
3. 2 × OD_cut_ < OD ≤ 4 × OD_cut_ = Moderate biofilm-former (MBF).
4. OD ˃ 4 × OD_cut_ = Strong biofilm-former.

In this work, sterile broth and non-biofilm-producing bacteria, *S. epidermidis* ATCC 12,228, were used as a negative control). Experiments with clinical isolates were performed in nonuplets. Nonuplets means that each isolate was inoculated in nine wells simultaneously and repeated three times. Methodology was carried out on different days; then, the OD values were averaged, and the SD was calculated.

### 2.6. Congo Red Agar (CRA) Assay

The protocol was based on Kaiser, T.D.L. et al., [24]. Briefly, the strains were cultured in TSB overnight and streaked into CRA plates. Then, the plaques were incubated at 37 °C in aerobic condition for 24 h. The production of black or brown colonies were considered as a positive strain for slime production. The red or pink colonies were considered as non-slime-producing strains, indicating weak or with no capacity to form a biofilm with our conditions. To evaluate the effect of NQo, we added the NQs directly to TSB, incubated overnight and then streaked on CRA plates as described above [25].

### 2.7. Assays to Determination of the Minimum Inhibitory and Bactericidal Concentrations of NQo

The inhibitory and bactericidal concentrations of NQo against *S. aureus* was determined following the guidelines of the CLSI [26] and the previous report by Rivera et al., [10]. Briefly, the overnight culture of *S. aureus* was diluted at a turbidity of 0.05 at OD600 and used in the presence or absence of chitosan nanoparticles (NQo). The NQo concentrations evaluated were from 14 to 1400 μg/mL; each assay was performed in triplicate. The cultures were incubated at 37 °C for 24 h in shaking conditions. After incubation, the OD600 of the culture was measured using an Infinite m200 Pro TECAN Plate Reader (Tecan AG Männedorf, Switzerland). The inhibitory concentrations were recorded as the lowest concentration that resulted in no visible growth of microorganisms after 24 h of incubation at 37 °C (no turbidity). To determine the MBC, a plate count of viable cells method (CFU) was determined, as described by Rivera et al., [10]. A volume of 1.0 mL of the culture exposed to NQo was inoculated in 9.0 mL of NaCl (0.9% *w*/*v*), and serial dilutions were made up to 10^−5^. The resulting bacterial suspension (1000 μL) was spread and plated onto a Tryptic Soy Agar plate and incubated at 37 °C for 24 h. After incubation, the CFUs that appeared on the Tryptic Soy Agar plate were counted, where the result was expressed as CFU/mL. The lowest concentration that killed 99.9% of the starting inoculum was defined as the MBC [27].

### 2.8. Disk Diffusion Test: Performance of NQo Versus Conventional Antibiotics

The agar diffusion test was used to compare the antimicrobial activity of NQo compared to conventional antibiotics on the survival of *S. aureus* strains isolated from MB. The assays were carried out according to the methodologies of Bauer et al., [28].

A volume of 0.1 mL of a bacterial suspension (~1 × 10^6^ CFU/mL) was inoculated into Mueller–Hinton agar plates (MHAP). Blank disks were loaded with 10 μg of NQo; these concentrations were equivalent to antibiotic disks concentrations, gentamicin (10 μg) and ampicillin (10 μg). Additionally, in this assay, disks were loaded with 2.5 and 5.0 μg of NQo. The MHAPs were incubated at 37° C for 24 h, and the inhibition was estimated by measuring the diameters of the areas with no bacterial growth and were compared with the inhibition area of gentamicin and ampicillin. This experiment was performed in triplicates.

### 2.9. Biofilm Inhibition by NQo

The biofilm inhibition capacity of NQo at sub-minimal inhibition concentrations (sub-MICs) was quantified using the crystal violet method; a 96-well polystyrene microtiter plate was used as the surface for the bacterial biofilm formation at 37 °C [10]. The *Staphylococcus aureus* culture was grown overnight and then was diluted until an OD600 of 0.05 was observed, which was used for biofilm production in the presence and absence of NQo. Diverse sub-MICs were added to the 96-well microtiter plate containing 250 μL of the *S. aureus* culture and incubated at 37 °C for 24 h. The planktonic cells were discarded after incubation and fixed in methanol, followed by staining with the crystal violet. The *S. aureus* biofilms were washed 3 times with water, re-suspended in glacial acetic acid (33% *v*/*v*), and measured at OD570 using the Infinite m200 Pro TECAN Plate Reader (Tecan AG Männedorf, Switzerland).

### 2.10. Statistical Analysis

All experiments in this study were carried out in triplicate. The figures and graphs shown in this report were elaborate using the program GraphPad Prism 6.0 (GraphPad Software Inc., San Diego, CA, USA), and all results obtained and indicated were presented as means ± standard deviation (SD). Statistical analysis of the data was performed using a one-way ANOVA followed by a Tukey a posteriori test. Differences with a *p* < 0.05 were considered statistically significant.

## 3. Results

### 3.1. Characterization of Chitosan Nanoparticles (NQo)

**Figure 1:**
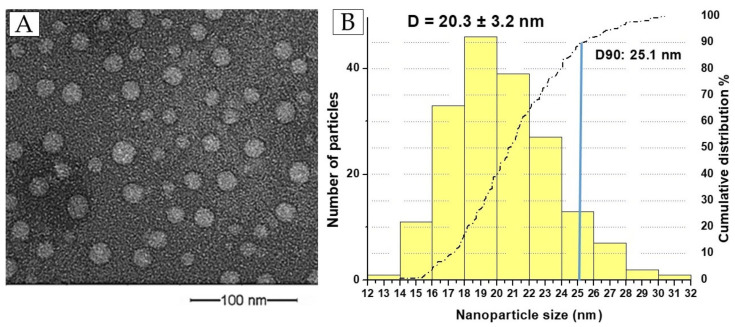
(**A**) Transmission electron microscopy (bright field) of NQo. (**B**) Histogram: NQo size distribution. (**A**) Transmission electron microscopy (TEM) of NQo. (**B**) Histogram: NQo size distribution. (blue line represents the D90 in cumulative analysis-dash dot line).

The morphology of the NQo was thoroughly characterized using transmission electron microscopy (TEM), as shown in Figure 1A. TEM imaging was conducted under bright-field conditions to ensure detailed visualization of the nanoparticles. The resulting micrographs reveal that the NQo exhibit a spherical shape with a mean size of 20.3 ± 3.2 nm. Additionally, these nanoparticles achieved a high particle yield (YP%) of 92.8 ± 1.3%, indicating the efficiency of the synthesis process. To further analyze the particle size distribution and its normal deviation, a histogram was generated using the ImageJ 1.52v software (Figure 1B). The cumulative analysis revealed that 90% of the particles had a size less than 25.1 nm (dash dot line). This approach provided a quantitative assessment of particle uniformity, a critical parameter for ensuring consistent behavior in biomedical applications.

The NQo exhibited a hydrodynamic diameter with an average size of 119.8 ± 2.6 nm (Table 1), as determined via dynamic light scattering (DLS). This measurement reflects the effective size of the nanoparticles in suspension, taking into account the solvation layer surrounding them. The polydispersity index (PDI) was recorded at 0.668 ± 0.009, indicating a moderate level of size distribution, which is critical for assessing the uniformity and consistency of the nanoparticle population. Furthermore, the zeta potential was measured at +55.4 ± 2.5, a value that signifies strong electrostatic repulsion between particles, contributing to their stability in suspension.

### 3.2. Biochemical and Molecular Characterization of S. aureus from Milk Samples

Of the fifteen samples obtained for this study, in a first stage, given the morphology observed under the microscope (spherical shape), cellular-grouping (grape-like clusters), staining affinity (Gram-positive), catalase and coagulase-positive assay, and hemolytic capacity in blood agar (β-hemolysis), six strains were selected. These strains were grown in Baird Parker and Mannitol Salt Agar, obtaining shiny black colonies that were round, had smooth edges, and were surrounded by the presence of pale-yellow halos of light around the colonies and round yellow colonies with smooth edges. The medium produced a color change from orange–red to yellow. DNA was extracted from these six strains to perform PCR with the aim of sequencing by amplification of the conserved ribosomal fraction 16S gene (rRNA). The results of this biochemical and molecular characterization evidenced the isolation of the *S. aureus* strains from the milk samples.

### 3.3. Characterization of Biofilm Formation Capacity (BFC) Using S. aureus Isolated from Milk Samples of Cows Diagnosed with BM

**Figure 2:**
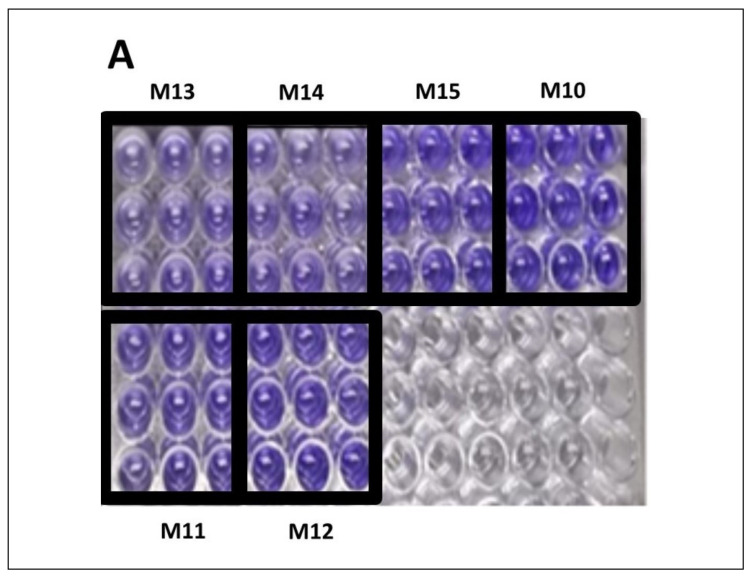

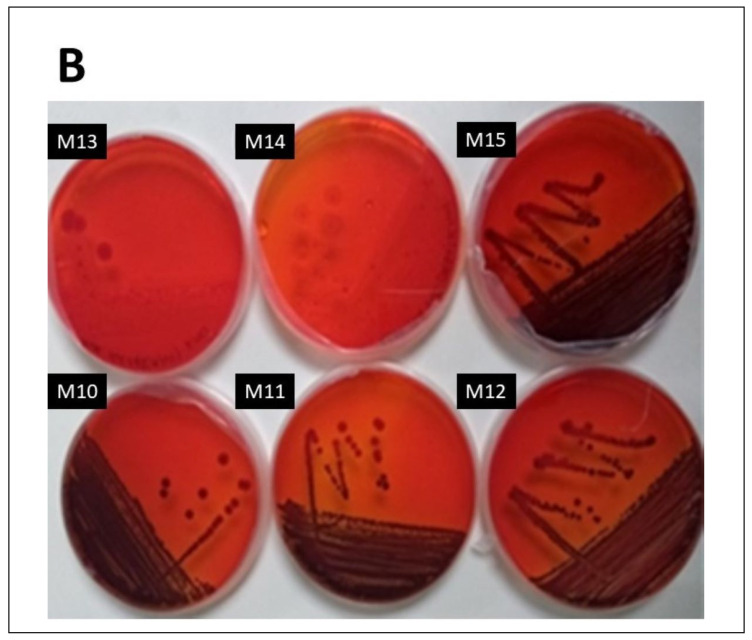
Characterization and quantification of BFC of *S. aureus* strain isolated from milk samples: (**A**) crystal violet assay and (**B**) Congo red agar assay.

After bacteriological isolation and biochemical and molecular characterization, in this study, six strains of *S. aureus* were obtained from milk samples of cows with BM. These strains and the nomenclature assigned (with M letter and correlative numbers from 10 to 15) to each of them are shown in Table 1. Subsequently, we evaluated the capacity of these isolated *S. aureus* strains to produce biofilm (Table 2 and Figure 2). The biofilm formation capacity (BFC) was determined via the biofilm production in the crystal violet (CV) assay [29], and the strains were classified and categorized using the calculation of the ODcut [10]. Also, we performed the Congo red agar (CRA) assay to detect slime production [24]. The six samples were able to produce biofilm with different capacities (Table 2), and four of them were also positive in the CRA assay (Figure 2B).

The BFCs of the strains M10 and M15 showed the highest capacity to form the biofilm, determined by the OD595 thrown in the cv test, with values of 0.325 ± 0.03 and 0.317 ± 0.02, respectively. These values when using the ODcut have been classified as strong biofilm-forming strains (S). Regarding the BFCs of M11, M12, and M13, these strains showed OD595 in the cv test of 0.293 ± 0.05, 0.298 ± 0.02, and 0.213 ± 0.03, respectively, showing an intermediate phenotype (I) in their ability to form the biofilm. Finally, strain M14, even though it showed the capacity for biofilm formation, showed a weak capacity (W) in the formation of the biofilm under the conditions tested. It is interesting that the methodological strategies used in this study, both the CV assay and the spread of the strains in the CRA medium, showed similar phenotypes, allowing for the specific classification of these isolated strains regarding their ability to produce biofilm in vitro.

### 3.4. Minimum Inhibitory Concentration and Minimum Bactericidal Concentration

The antimicrobial capacity of NQo was verified, challenging the NQo against the isolated strain of *S. aureus*. Observing Table 3, it is indicated that the values of the minimum inhibitory concentration (MIC) and minimum bactericidal concentration (MBC) of NQo against *S. aureus* were 128 μg/mL and 250 μg/mL, respectively. This assay was verified using absorbance measurements to 600 nm and the viable cell count method, with which it was quantified.

### 3.5. Disk Diffusion Test and Comparison of NQo to Conventional Antibiotics

The sensitivity of the six characterized strains of *S. aureus* (M10 to M15) against the gold standard antibiotics used for the treatment of BM was evaluated as a first strategy. The strains were confronted with disks loaded with ampicillin (Amp 10 μg) and gentamicin (CN 10 μg) via the agar plate diffusion technique. Table 3 shows that all the evaluated strains were sensitive to both antibiotics. A higher sensitivity was observed against penicillin than gentamicin in identical concentrations in all the strains evaluated. All these strains were 1.7 times more sensitive to Amp than CN, where the average inhibition halo was 22.05 ± 1.17 mm for Amp and 12.97 ± 1.29 mm for CN.

In relation to the results of the antibiogram and those obtained in the phenotypic and biofilm formation capacity (Table 2 and Figure 2), it was decided to carry out the following tests with the M15 *S. aureus* strain, since it is one of the strains with a strong phenotype in the ability to form biofilm.

**Figure 3:**
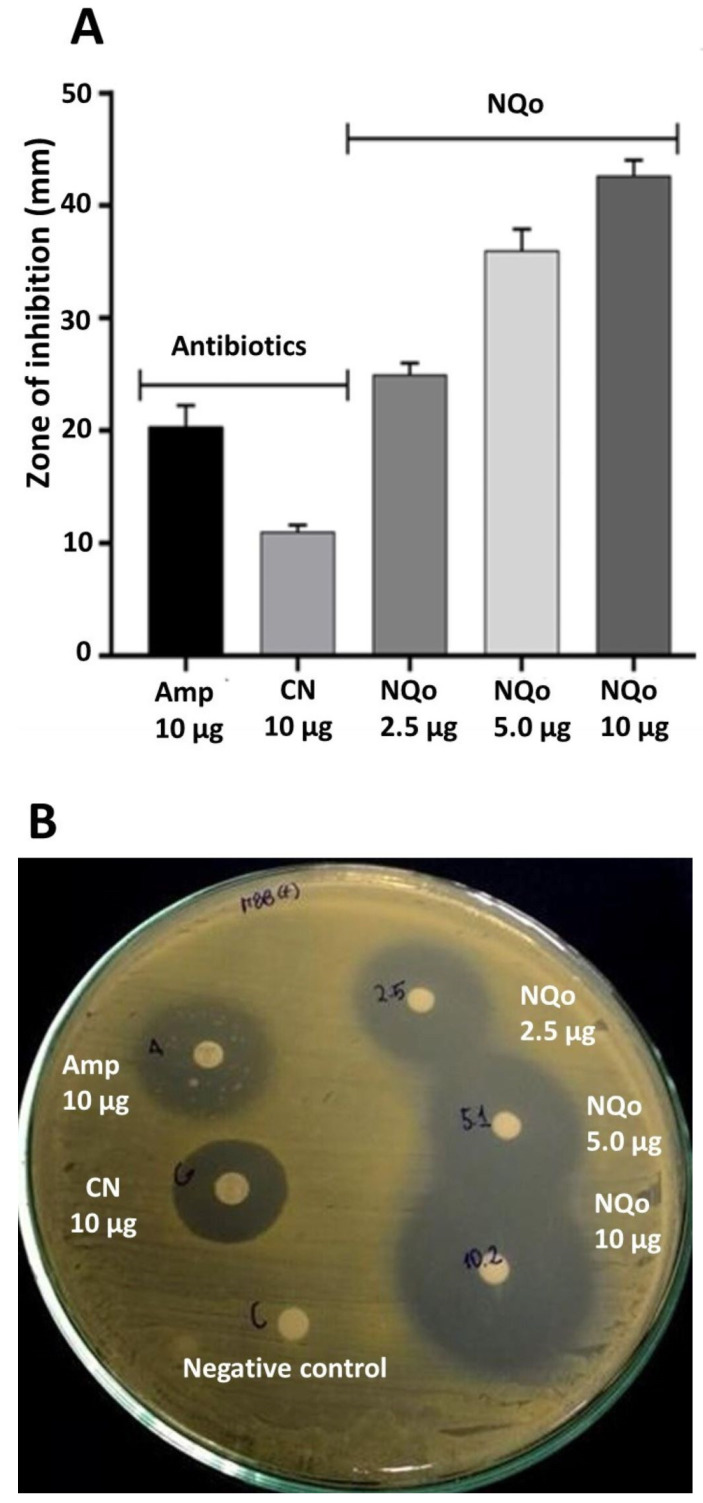
Growth inhibition zone of NQo on *Staphylococcus aureus* (M15 strain): (**A**) graphical representation of comparative inhibition zone between conventional antibiotics and NQo on three different concentrations; (**B**) antibiogram photographs on agar. Amp = ampicillin, CN = gentamicin, NQo = chitosan nanoparticles.

Another technique to evaluate the antimicrobial capacity of the NQo against strain M15 of *S. aureus* was the disk diffusion test (Kirky–Bauer method) and comparing the effectiveness of the inhibition of these nanoparticles with respect to the previously evaluated antibiotics, Amp and CN, in equivalent concentrations (10 μg). Additionally, in this same assay, evaluating concentrations lower than the antibiotic disks in 50% and 75% lower concentrations of nanoparticles was considered, that is, 5 μg and 2.5 μg. The results are shown in Figure 3A,B. It was observed that the antibiotic Amp showed a diameter of inhibition of 20.35 ± 1.88, and CN displayed an inhibition of 11.04 ± 0.56, while the zone of growth inhibition evidenced in the loaded disks with 10 μg of NQo was 46.44 ± 1.38 mm. These results show the high capacity of these nanoparticles in antimicrobial activity compared to conventional antibiotics. When comparing NQo with AMp, these were 2.1 times more powerful, showing a 52.3% greater effectiveness in concentrations equivalent to 10 μg. A greater effect was observed when comparing it with the CN: the nanoparticles resulted in a 3.9 times greater effect, with a 74% higher effectiveness in identical concentrations tested. On the other hand, when lower concentrations of NQo were tested, compared to commercial disks loaded with antibiotics (Amp or CN), it was shown that when using a 50% lower concentration of nanoparticles (NQo 5 μg), a zone of growth inhibition of 36.01 ± 1.88 mm was observed in this strain. This concentration of NQo loaded in the disks was 1.8 and 3.3 times more powerful in antimicrobial activity with 43.5% and 69.3% effectiveness compared to Amp and CN, respectively. Interestingly, it was observed that when 2.5 μg were loaded on the disks, that is, a 75% lower load than commercial antibiotic disks, a zone of inhibition of 24.99 ± 1.01 mm was observed in the *S. aureus* strain. The inhibition with this concentration of NQo was 1.2 and 2.3 times more powerful than Amp and CN, respectively.

### 3.6. NQo Reduce Biofilm Formation of S. aureus

**Figure 4:**
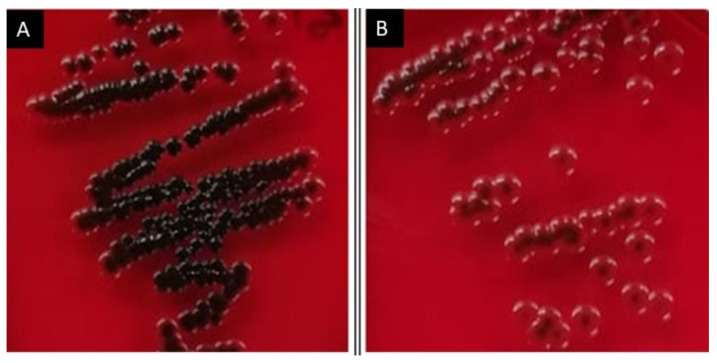
NQo change the phenotype of *S. aureus* in CRA: (**A**) sample growth in normal culture medium; (**B**) sample growth in normal culture medium but exposed at 64 µg/mL of NQo.

**Figure 5:**
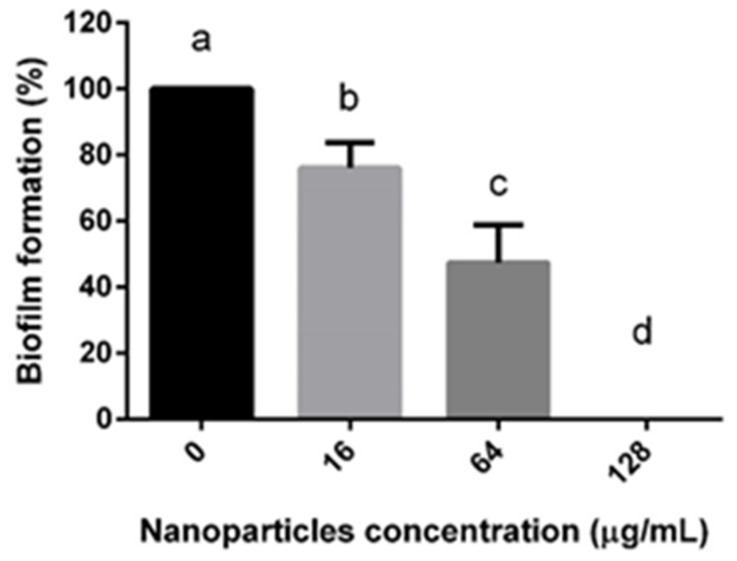
Crystal violet assay with varying NQo concentrations on *S.aureus* (M15 strain): a, control; b, NQo 16 µg/mL; c, NQo 64 µg/mL; d, NQo 128 µg/mL.

To evaluate the effect of the NQo over biofilm formation, we selected one isolated *S. aureus* strain that produced a positive phenotype in the CRA assay and had a strong biofilm formation capacity, corresponding to isolated M15. One approximation was co-incubating 64 μg/mL of NQo (equivalent to 50% of the MIC) in the normal culture medium and then streaking it on the Congo red agar. The culture exposed to NQo shifted its strong positive phenotype in the CRA assay (dark colonies) towards red colonies with brown centers (Figure 4), indicating that NQo affect the production of slime in this strain. Finally, we used the crystal violet assay adding different NQo concentrations (Figure 5). The measurements show that increasing the concentration of NQo in the medium decreases the percentage of biofilm formation compared with the control condition, supporting that NQo reduce biofilm formation of isolated *S. aureus*.

## 4. Discussion

### 4.1. Characterization of Chitosan Nanoparticles (NQo)

The physicochemical characterization of NQo is presented in Figure 1, showcasing the size measurements obtained through dynamic light scattering (DLS) and transmission electron microscopy (TEM). The DLS analysis revealed larger size values compared to those determined with the TEM. This discrepancy arises from the fundamental differences in measurement techniques. TEM provides direct visualization of dry samples, capturing the physical dimensions of individual particles in a static state. In contrast, DLS measures the hydrodynamic diameter of nanoparticles in suspension, which includes the particle core, its solvation layer, and any associated hydration shell [22,30]. This measurement is based on the random motion of particles in a liquid medium, known as Brownian motion. As previously reported [14,21], these nanoparticles exhibit a tendency to agglomerate during dynamic light scattering (DLS) measurements. Therefore, it is essential to optimize the sample concentration and subject the sample to sonication prior to analysis. This challenge can be mitigated by incorporating the nanoparticles into a viscous medium, such as a hydrogel [31], which would promote colloidal stabilization of the particles and facilitate the application of the final formulation to the target tissue in infected animals. A viscous intramammary injectable remains the most commonly employed method for delivering antimicrobials in the treatment of mastitis [32].

The Z-potential analysis revealed a value exceeding +20 mV, indicating a strongly positive surface charge on the nanoparticles. This high zeta potential is a critical factor for ensuring the stability of the nanoparticles in suspension, as it creates significant electrostatic repulsion between particles, reducing the likelihood of aggregation. Stable, well-dispersed nanoparticles are essential for maintaining their structural integrity over extended periods, which is particularly important for biomedical and industrial applications. When considered alongside the nanoparticles’ optimal size and moderate polydispersity index (PDI), the significant zeta potential further underscores the versatility and reliability of NQo. These combined physicochemical properties make them highly suitable for applications requiring consistent and predictable behavior. For instance, in drug delivery systems, these characteristics facilitate precise targeting and controlled release. In antimicrobial treatments and biofilm inhibition, the stability and uniform dispersion of nanoparticles enhance their efficacy by allowing them to interact more efficiently with bacterial cells or biofilm structures. These features collectively position NQo as a promising platform for innovative therapeutic and industrial solutions.

### 4.2. Antimicrobial Capacity of NQo

#### 4.2.1. The Minimum Inhibitory Concentration (MIC) and the Minimum Bactericidal Concentration (MBC)

The MIC and MBC ratios of NQo against all strains of *S. aureus* assay in this work were found to be 128 μg/mL and 250 μg/mL, respectively (Table 3). The antimicrobial capacity (bacteriostatic and bactericidal) of these kinds of nanoparticles (NQo) was important to determine their efficacy against different strains of *S. aureus*. Different data reported in the literature indicate a bacteriostatic activity (MIC) for chitosan nanoparticles against *S. aureus* covering concentration ranges from 128 to 4096 µg/mL [33], evidencing that our results require low concentrations to obtain an effect on the proliferation of *S. aureus*.

Vimal et al., [34] reported MICs of NQo for the pathogens *Pseudomonas aeruginosa*, *Klebsiella pneumoniae*, *Salmonella typhimurium*, and *Proteus vulgaris*, obtaining 128 µg/mL, 512 µg/mL, 128 µg/mL, and 64 µg/mL, respectively.

The MIC values of NQo for the *Staphylococcus aureus* strains isolated from bovine mastitis (BM) cases ranged between 200 and 400 μg/mL [35]. Comparatively, another study evaluating the antimicrobial activity of low-molecular-weight chitosan against BM pathogens reported higher MIC and minimum bactericidal concentration (MBC) values of 800 μg/mL and 1600 μg/mL, respectively [36]. Notably, for BM-related *Pseudomonas aeruginosa* strains, NQo demonstrated MIC values ranging from 280 to 400 μg/mL, highlighting its broad-spectrum antimicrobial potential against diverse pathogens associated with BM [6]. Kucharska et al. [37] reported that the bactericidal activity of chitosan molecules is influenced by their molecular weight and degree of deacetylation. Specifically, the bactericidal capacity is inversely proportional to molecular weight and directly proportional to the degree of deacetylation. Low-molecular-weight chitosan molecules exhibit the ability to penetrate bacterial cells and electrostatically interact with negatively charged intracellular components. This interaction disrupts the molecular machinery of the genetic material, such as DNA and/or mRNA, ultimately impairing essential cellular processes and contributing to bacterial cell death [37].

#### 4.2.2. Disk Diffusion Test and Comparison of NQo to Conventional Antibiotics

Table 4 presents the average inhibition zones produced by *S.aureus* strains isolated from bovine mastitis samples when exposed to ampicillin and gentamicin. Ampicillin demonstrated greater antibacterial activity compared to gentamicin. Notably, the antibacterial efficacy of NQo was observed to be twice as effective as conventional antibiotics, as evidenced by a twofold increase in the size of the growth inhibition zones (Figure 3). Furthermore, *S. aureus* strains were found to exhibit resistance to both ampicillin and gentamicin, while remaining highly sensitive to NQo, highlighting its potential as a superior therapeutic agent. Similar findings regarding the antimicrobial susceptibility of *S.aureus* to ampicillin and gentamicin have been reported in studies conducted on dairy farms [38,39]. These results align with another investigation on isolates from subclinical bovine mastitis, where *S. aureus* demonstrated resistance to ampicillin but sensitivity to gentamicin. The study further revealed that 75% and 61% of weak and moderate biofilm producers, respectively, were resistant to antimicrobials, compared to only 12.5% of strong biofilm producers. This suggests that antimicrobial-resistant isolates are more likely to form weak to moderate biofilms, highlighting a potential link between biofilm formation capacity and resistance profiles [40].

#### 4.2.3. NQo Reduce Biofilm Formation of *S. aureus*

Biofilm formation represents a significant challenge in modern antibacterial therapy. The ability of bacteria to form biofilms confers several advantages to the pathogen, primarily by creating a protective barrier that inhibits the penetration of antimicrobial agents. This barrier also impedes the host immune system’s ability to effectively eliminate the pathogens, thereby increasing their persistence and resistance to treatment [41,42]. The ability of NQo to inhibit the formation of biofilms by *S. aureus* was evaluated by quantifying the biofilm formed by the M15 sample in the presence of different sub-MICs of NQo. As shown in Figure 4 and Figure 5, the results indicated a great performance of the NQo against the inhibition of biofilm formation; the higher the nanoparticle concentration, the greater the inhibition. In the literature, the biofilm inhibitory effect of NQo *on S. aureus* is reported. The researchers [43,44] also obtained a percentage greater than 50% inhibition in the formation of biofilms treated with NQo. On the other hand, authors have observed a significant reduction in the development of biofilms when the bacterial strains were treated with 1/4 MIC of NQo in *Pseudomonas aeruginosa* cultures, decreasing the formation of biofilms by 50% [45]. The bacterial genera Staphylococcus and Pseudomonas, particularly the species *Staphylococcus aureus* and *Pseudomonas aeruginosa*, exhibit the capability to produce extracellular polymeric substances. These polymeric molecules play a crucial role in facilitating bacterial adhesion to host cells and the formation of a polymeric matrix, which constitutes the structural foundation of biofilms. This complex extracellular network enhances the bacteria’s ability to evade the host immune system and resist conventional antibiotic treatments, contributing to their persistence and pathogenicity [46].

The antibiofilm activity of nanoparticles may stem from their ability to inhibit the growth of extracellular polymeric substances (EPS). By disrupting EPS production, nanoparticles interfere with the formation and stabilization of biofilms, ultimately reducing biofilm development and bacterial adherence [47]. The antibiofilm activity of chitosan can be attributed to its ability to disrupt bacterial communication systems, which are essential for coordinating processes like biofilm formation. As a cationic polymer, chitosan interacts with negatively charged microbial cell membranes, disrupting their functionality and interfering with microbial signaling pathways. This interaction reduces the secretion of signaling molecules, thereby inhibiting the production of virulence factors and slowing biofilm formation. This mechanism highlights chitosan’s potential as an effective agent for controlling biofilm-associated infections [48].

## 5. Conclusions

In this work, the ionic gelation process with sodium tripolyphosphate allowed for the elaboration of chitosan polymeric nanoparticles with stable physicochemical properties. These NQo were tested against a strain of *S. aureus* isolated from milk samples obtained from specimens diagnosed with BM from the southern zone of Chile (Los Muermos, X Region), a sector characterized as a large dairy producer. These strains were identified through biochemical tests on agar and molecular sequencing assays. *S. aureus* has been indicated as the most prevalent strain related to this disease in cattle. This work studied the behavior of these strains, particularly when confronted with NQo, which may not necessarily be representative of all dairies at a national or global level; however, it is an interesting approach to communicate the effectiveness of this type of nanoparticles in any of the bacterial etiological agents responsible for this infection in cows around the world. Here, the antimicrobial activity was evaluated, including the assays of minimum inhibitory and bactericidal concentrations, the disk diffusion test to evaluate the performance of NQo versus conventional antibiotics, and the inhibition of biofilm formation. These NQo showed excellent performance relative to their antimicrobial capacity, inhibiting bacterial proliferation and allowing for negative modulation of biofilm production in these *S. aureus* strains and, interestingly, allowing biofilm inhibition even when sublethal concentrations were tested. The performance of the NQo relative to the antimicrobial capacity compared to conventional antibiotics, ampicillin and gentamicin, strongly suggest that these nanoparticulate polymeric constructs could be an attractive alternative for the control, prevention, and/or treatment of bovine mastitis. Future research will focus on the development of an intramammary injectable formulation utilizing a viscous hydrogel, aimed at enhancing product retention and ensuring the colloidal stability of the nanoparticles. Further in vitro and in vivo pharmacokinetic studies are warranted to elucidate the distribution profile of the nanoparticles, thereby ensuring their efficacy and safety. It is also necessary to elucidate the possible molecular mechanisms on the regulation of the expression of genes related to biofilm formation. The use and application of new molecules, in particular, the use of nanotechnology of natural polymers, which show adequate antimicrobial activity, superior to conventional antibiotics, is increasingly urgent due to the imminent threat of superbugs spread and the non-antibiotic treatment capacity, resulting in a real threat to animal and human health.

## Figures and Tables

**Table 1:** Dynamic light scattering (DLS) characterization of chitosan nanoparticles (NQo).

Sample	Z-Average (nm)	PDI	Zeta Potential (ζ) (mV)
NQo-1	122.2	0.678	+52.6
NQo-2	120.0	0.666	+56.3
NQo-3	117.1	0.661	+57.3
Average	119.8 ± 2.6	0.668 ± 0.009	+55.4 ± 2.5

**Table 2:** Biofilm formation capacity of isolated *S. aureus* samples determined using crystal violet (CV) assay, ODcut method, and Congo red agar (CRA).

Strain of *S. aureus* Sample Identification	Biofilm Quantification (OD CV Assay)	Biofilm Formation Capacity (ODcut Method)	Phenotype CRA
M10	0.325 ± 0.03	Strong	Positive
M11	0.293 ± 0.05	Intermediate	Positive
M12	0.298 ± 0.02	Intermediate	Positive
M13	0.213 ± 0.03	Intermediate	Negative
M14	0.164 ± 0.05	Weak	Negative
M15	0.317 ± 0.02	Strong	Positive

**Table 3:** Susceptibility of *S. aureus* to NQo.

Strains	MIC (μg/mL)	MBC (μg/mL)	MIC/MBC
M10	128	250	1.95
M11	128	250	1.95
M12	128	250	1.95
M13	128	250	1.95
M14	128	250	1.95
M15	128	250	1.95

**Table 4:** Antibiogram of *S. aureus* strains isolated from milk samples of cows diagnosed with BM against ampicillin and gentamicin.

	Zone of Inhibition (mm)	
*S. aureus* strain	Amp	CN
M10	22.18 ± 1.34	12.32 ± 0.78
M11	23.85 ± 1.66	14.79 ± 0.98
M12	22.45 ± 2.02	13.12 ± 0.65
M13	21.29 ± 1.45	12.67 ± 0.34
M14	22.18 ± 1.22	13.88 ± 0.63
M15	20.35 ± 1.88	11.04 ± 0.56

## Data Availability

No new data were created or analyzed in this study. Data sharing is not applicable to this article.
